# In-situ analysis of continuous cooling precipitation in Al alloys by wide-angle X-ray scattering

**DOI:** 10.1080/14686996.2020.1739554

**Published:** 2020-04-02

**Authors:** Christian Rowolt, Hannes Fröck, Benjamin Milkereit, Michael Reich, Wolfgang Kowalski, Andreas Stark, Olaf Keßler

**Affiliations:** a Chair of Materials Science, Faculty of Mechanical Engineering and Marine Technology, University of Rostock, Rostock, Germany; b Competence Centre °CALOR, Department Life, Light & Matter, Faculty of Interdisciplinary Research, University of Rostock, Rostock, Germany; c Institute of Materials Research, Helmholtz-Zentrum Geesthacht, Geesthacht, Germany

**Keywords:** Wide-angle X-ray scattering (WAXS), HEXRD, quench sensitivity, continuous cooling precipitation, EN AW-7150, EN AW-6082, differential scanning calorimetry (DSC), 504 X-ray / Neutron diffraction and scattering, 106 Metallic materials, 302 Crystallization / Heat treatment / Crystal growth

## Abstract

The aim of this work is to investigate quench induced precipitation during continuous cooling in aluminium wrought alloys EN AW-7150 and EN AW-6082 using in situ synchrotron wide-angle X-ray scattering (WAXS). While X-ray diffraction is usually an ex situ method, a variety of diffraction patterns were recorded during the cooling process, allowing in situ analysis of the precipitation process. The high beam energy of about 100 keV allows the beam to penetrate a bulk sample with a 4 mm diameter in a quenching dilatometer. Additionally, the high intensity of a synchrotron source enables sufficiently high time resolution for fast in situ cooling experiments. Reaction peaks could be detected and compared with results from differential scanning calorimetry (DSC) by this method. A methodology is presented in this paper to evaluate WAXS data in a way that is directly comparable to DSC-experiments. The results show a high correlation between both techniques, DSC and WAXS, and can significantly improve continuous cooling precipitation diagrams.

## GRAPHICAL ABSTRACT

**Figure:**
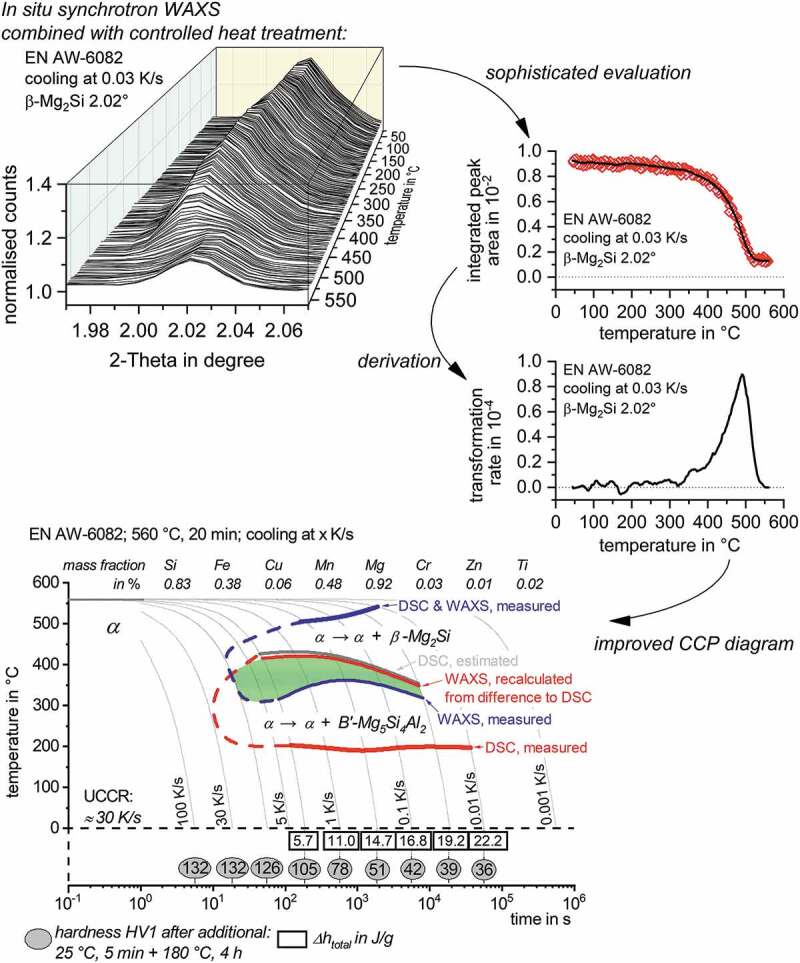

## 1 Introduction

The mechanical properties of several aluminium alloys are mainly adjusted by age hardening heat treatment via the mechanism of particle strengthening. The age hardening process consists of three steps – solution annealing, quenching and ageing [1]. During solution annealing at elevated temperatures, alloying elements are dissolved in the solid Al matrix crystal. A supersaturated solid solution originates after fast quenching to room temperature. During the ageing step, nanoscale secondary particles are precipitated that hinder dislocation movement and increase the strength of the alloy.

If, during the quenching step, the alloy is cooled slower than the upper critical cooling rate, detrimental coarse quench induced precipitation might occur. These quench induced precipitates frequently cause reduced strength. This phenomenon is typically called ‘quench-sensitivity’ [2–6]. The quench-sensitivity of precipitation hardening aluminium wrought alloys has been intensively studied by differential scanning calorimetry (DSC) in the last decade [7–12]. DSC is a powerful in situ method that is capable of detecting cooling precipitation kinetics over a very wide range of cooling rates and allows evaluating the overall precipitation enthalpies [13]. One limitation of DSC is that it detects the sum of all ongoing reactions. As the relevant precipitation reactions frequently overlap, DSC often does not allow the separation of single reactions or the ability to evaluate their specific precipitation enthalpies [14]. Sometimes, particularly for high concentration alloys, the overlapping of single reactions is so severe that DSC detects just one broad peak [15].

The maximum strength after ageing can typically be achieved when quench induced precipitation is completely suppressed. The kinetic evolution of quench induced precipitation is described in continuous cooling precipitation (CCP) diagrams, which have been investigated in the authors’ previous works for EN AW-7150 [8,9] and EN AW-6082 [10,11]. To obtain such CCP diagrams the kinetic analysis from DSC needs to be complemented by sophisticated microstructural analysis. A wide range of cooling rates is relevant for quench induced precipitation; the dimensions of those precipitates might vary from several 10 µm down to just a few nm [16]. Therefore, a range of methods needs to be applied to analyse the microstructural changes caused by quench induced precipitation. Typical methods for imaging the precipitates are optical microscopy (OM), scanning electron microscopy (SEM), and transmission electron microscopy (TEM) [11,17,18]. Usually these methods are applied ex situ, after the cooling is completed.

Another option for detecting quench-induced precipitates is through X-ray scattering effects, i.e. by applying small- and/or wide-angle X-ray scattering (SAXS/WAXS) measurements while cooling relevant alloys (examples: SAXS [19–21], WAXS [22,23]). The SAXS detection limit, in terms of particles size, depends on the smaller angle-resolution and, therefore, is dependent on the distance between the detector and the sample [24]. However, SAXS is typically restricted to the detection of very small precipitates with dimensions below about 100 nm [25–27]. This is a severe limitation for detecting quench-induced precipitation as several of the quench-induced particles – particularly those of the equilibrium phases – are much larger. Besides, ultra-small-angle X-ray scattering (USAXS) exists, enabling static and kinetic investigations of particles with dimensions down to micrometres, at a time scale shorter than milliseconds [28]. If those instruments would allow controlled heat treatment of the samples including controlled cooling, they might be suitable for analysis of precipitation during continuous cooling.

WAXS also allows the detection of phases distributed in much larger particles and beyond that yields structural information, which potentially allows to follow the precipitation of specific phases. The suitability of WAXS for analysing the phase transformation kinetics in metallic materials was shown in [29,30], for example. It is, therefore, one potential option for the in situ detection of quench induced precipitation. In contrast to DSC, WAXS is sensitive to the occurrence of individual crystal structures and, thus, correlating DSC and WAXS is a promising approach to get more insight into the overlapping of quench induced precipitation reactions.

The idea of this work, therefore, is to use in situ synchrotron transmission WAXS, or high-energy X-ray diffraction (HEXRD), and its sensitivity to the appearance of specific crystal structures to determine the characteristic precipitation temperature ranges. It is aimed to correlate WAXS and DSC to enable assignment of specific quench induced phases to certain DSC peaks for the aluminium wrought alloys EN AW-7150 and EN AW-6082. A methodology is presented to obtain is-situ WAXS data during continuous cooling. Moreover, the suggested WAXS data analysis allows direct comparison to DSC cooling curves.

## 2 In situ cooling WAXS experiments

### 2.1 Experimental setup

**Figure 1.**
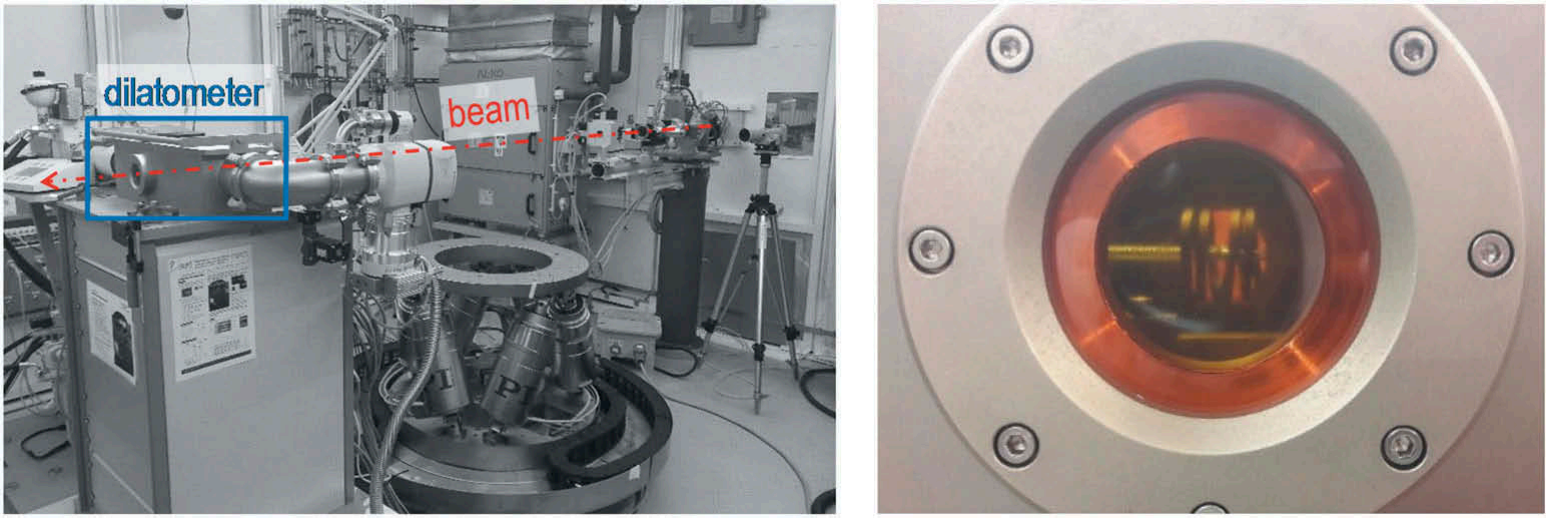
Experimental setup showing the beam schematic and the modified quenching dilatometer with an entrance/exit window

In situ transmission WAXS experiments were carried out at the DESY synchrotron facility in Hamburg, Germany (Deutsches Elektronen-Synchrotron). The high energy materials science (HEMS) beamline P07 at PETRA III, which provides tunable photon energies in the 30–200 keV range [31], was used. The high beam energy, in this case about 100 keV, allows the beam to penetrate a bulk metallic sample with a 4 mm diameter and the high intensity of a synchrotron source enables a sufficiently high time resolution for fast in situ cooling experiments. For details concerning X-ray diffraction, the reader is referred to [32]. To enable in situ analysis of phase transformations during controlled heat treatment, a modified quenching dilatometer, Bähr DIL805 A/D, equipped with windows for X-ray beam transmission was installed in the beamline, see Figure 1. The samples were heated inductively inside a vacuum chamber to the solution annealing temperature and, after complete soaking, were gas quenched in a controlled way. The induction coil used was adjusted to the needs of the beamline, allowing beam transmission, as can be seen in Figure 1.

A digital X-ray flat panel detector, PE XRD 1621 produced by PerkinElmer Inc., was used to record Debye-Scherrer diffraction rings. The detector (not shown in Figure 1, about 1.5 m behind the exit window in the beam direction) has the following specifications:

- detector size 410 x 410 mm
- resolution200 x 200 µm
- array2048 x 2048 pixels
- frame rate15 fps

### 2.2 Materials

Cylindrical aluminium alloy EN AW-7150 samples (Ø 4 × 10 mm) were cut from the centre layer of a commercially produced 80 mm thick plate. The initial condition of these samples was solution treated and water quenched. The samples of EN AW-6082 (Ø 4 × 10 mm) were prepared from a 10 mm thick commercial aluminium alloy sheet supplied in T651-condition. The chemical composition of the investigated alloys analysed by optical emission spectroscopy (OES) in Table 1 is compared to the standard compositions according to EN 573–3.

**Table 1.** Mass fractions of alloying elements in the investigated aluminium wrought alloys

mass fraction in %		Si	Fe	Cu	Mn	Mg	Cr	Zn	Ti	Zr
EN AW-7150	EN 573-3	<0.12	<0.15	1.9–2.5	<0.1	2.0–2.7	<0.04	5.9–6.9	<0.06	0.08–0.15
	OES	0.02	0.05	2.04	0.04	2.15	<0.01	6.33	0.01	0.12
EN AW-6082	EN 573-3	0.7–1.3	<0.5	<0.1	0.4–1.0	0.6–1.2	<0.25	<0.2	<0.1	-
	OES	0.83	0.38	0.06	0.48	0.92	0.03	0.01	0.02	-

### 2.3 Time-temperature course of the experiments

**Figure 2.**
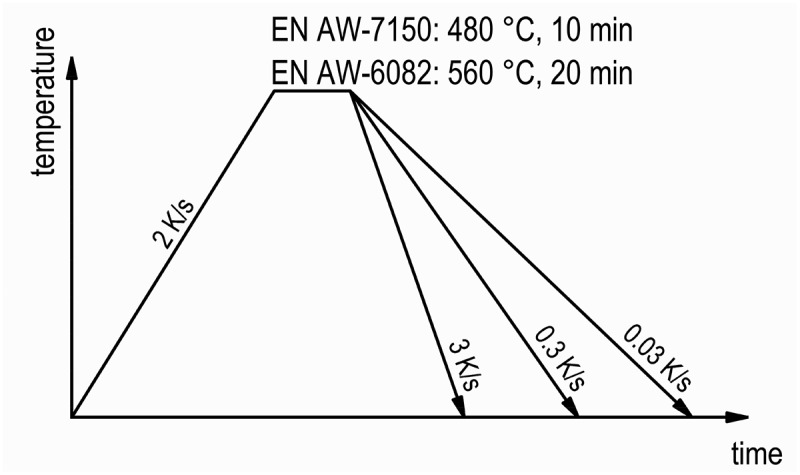
Schematic presentation of the time-temperature-profiles for the experiments

Continuous cooling experiments were performed to analyse the precipitation kinetics of EN AW-7150 and EN AW-6082 using in situ WAXS. Therefore, the samples were heated up to solution annealing temperature at 2 K/s. Solution annealing was done at 480°C for 10 min for EN AW-7150 and at 560°C for 20 min for EN AW-6082. For WAXS, three different cooling rates were chosen, 0.03 K/s, 0.3 K/s and 3 K/s, allowing correlation with existing DSC data [8,10]. A schematic time-temperature-profile can be seen in Figure 2. The detector allows capturing diffraction images every 0.1 s. To reduce the amount of data and improve the signal-to-noise ratio, the counts within a certain period of time were added up. The related periods were adjusted to the cooling rate, resulting in temperature steps of about 1 K for 0.03 K/s, 4 K for 0.3 K/s and 15 K for 3 K/s. The sample temperature at the end of the time step was used as the associated characteristic temperature. All experiments were done in vacuum and argon was used as the quenching medium.

## 3 WAXS raw data treatment and basic evaluation

### 3.1 WAXS raw data acquisition and transformation into 2D diffractograms

**Figure 3.**
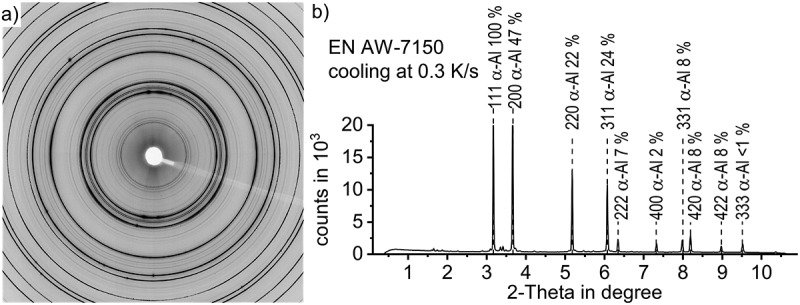
(a) diffraction pattern after cooling EN AW-7150 to room temperature at 0.3 K/s, as recorded by the imaging plate detector, (b) diffraction-data converted into a diffractogram at room temperature after cooling EN AW-7150 at 0.3 K/s

As raw data, the imaging plate detector captures the diffracted X-rays (tiff file format), appearing as characteristic Debye-Scherrer diffraction rings in Figure 3(a). Each concentric ring represents the intensity of diffraction caused by a specific crystal component, in particular to one of its specific lattice plane distances. Those rings, thus, can typically be attributed to a certain phase. The bright area in the centre is an artefact caused by a beam stop and the shadow ranging from the centre towards 4 o´clock was caused by fixing the beam stop in front of the plate detector.

In the first step of the data treatment, a rotational integration along the concentric rings was done using the software FIT2D and following Hammersley et al.’s [33] method. In this process the tiff files were converted into ASCII-format diffractograms containing the recorded counts versus diffraction angles 2-Theta for each temperature step, as shown in Figure 3(b). The required parameters implemented in FIT2D were as follows:

- calibrantlanthanumhexaboride (LaB_6_)
- beam centre 1027.558 x 1008.021pixels (205.512 x 201.604 mm)
- sample-to-detector distance 1544.026 mm
- wavelength 0.012958 nm
- tilt plane rotation angle -96.182°
- tilt angle -0.255°.

### 3.2 Phase identification via specifically selected diffraction peaks

The peaks within the integrated diffraction pattern can be identified using the DIFFRACplus PDFMaint database 15.0.0.0 (Bruker-AXS 1996–2009 [34],). Therefore, characteristic 2-Theta angles of all relevant lattice-plane distances were calculated using Bragg´s equation:

2⋅d⋅sinθ=n⋅λ

- distance between two atomic lattice planes *d*
- beamline wavelength λ = 0.012958 nm
- positive integer indicating the diffraction order *n* = 1.

Figure 3(b) shows the diffraction pattern of EN AW-7150 cooled from solution annealing at 0.3 K/s to room temperature, indicating the aluminium peaks and their relative peak height with respect to the peak of highest intensity in the particular lattice in %. From the literature, it is known that relevant phases for quench induced precipitation in EN AW-7150 include S-Al_2_CuMg precipitates and isostructural variants of η-Mg(Zn,Cu,Al)_2_ [16]. The structure of the latter are all believed to be based on the equilibrium C14 polytype of the Laves phase [35] and the Cu and Al contents have been discussed in several studies [36,37]. Selected 2-Theta angles for the characteristic crystal structure features of α-Al (face-centered-cubic, fcc), η-MgZn_2_ (hexagonal, a = 0.521 nm, c = 0.860 nm [1],) and S-Al_2_CuMg (orthorhombic, a = 0.400 nm, b = 0.923 nm, c = 0.714 nm [1],) phases with high diffraction intensities are shown in Table 2, while Table 3 gives this information for the β-Mg_2_Si phase (fcc, a = 0.639 nm [1],).

**Table 2.** Characteristic d-spacing, lattice parameters hkl and calculated 2-Theta angles specific to the applied wave-length (λ = 0.012958 nm) with associated intensities for the structures of α-Al (fcc, a = 0.405 nm, η-MgZn_2_ (hexagonal, a = 0.521 nm, c = 0.860 nm)and S-Al_2_CuMg (orthorhombic, a = 0.400 nm, b = 0.923 nm, c = 0.714 nm)

d in Å	2-Theta in degree	hkl	Intensity in %	d in Å	2-Theta in degree	hkl	Intensity in %	d in Å	2-Theta in degree	hkl	Intensity in %
2.338	3.18	111	100	4.522	1.64	100	59	4.620	1.61	020	90
2.024	3.67	200	47	4.284	1.73	002	33	3.880	1.91	021	14
1.431	5.19	220	22	3.999	1.86	101	32	3.570	2.08	002	30
1.221	6.08	311	24	2.414	3.08	103	40	3.270	2.27	111	60
1.169	6.35	222	7	2.230	3.33	112	85	2.829	2.62	022	18
1.012	7.34	400	2	2.186	3.40	201	100	2.561	2.90	112	60
0.929	8.00	331	8	2.142	3.47	004	31	2.311	3.21	131	100
0.906	8.20	420	8	1.999	3.71	202	44	2.200	3.38	041	30
0.827	8.99	422	8	1.936	3.84	104	16	2.016	3.68	132	30
0.780	9.53	333	<1	1.773	4.19	203	21	1.999	3.71	113	90
…	…	…	…	…	…	…	…	…	…	…	…
α-Al				η-MgZn_2_				S-Al_2_CuMg

**Table 3.** Characteristic d-spacing, lattice parameters hkl and calculated 2-Theta angles specific to the applied wave-length (λ = 0.012958 nm) with associated intensities for the structure of β-Mg_2_Si (fcc, a = 0.639 nm)

d in Å	2-Theta in degree	hkl	Intensity in %
3.668	2.02	111	41
3.176	2.34	200	12
2.246	3.31	220	100
1.915	3.88	311	15
1.834	4.05	222	2
1.588	4.68	400	13
1.457	5.10	331	6
1.420	5.23	420	3
1.296	5.73	422	21
1.222	6.08	511	4
…	…	…	…
β-Mg_2_Si			

**Figure 4.**
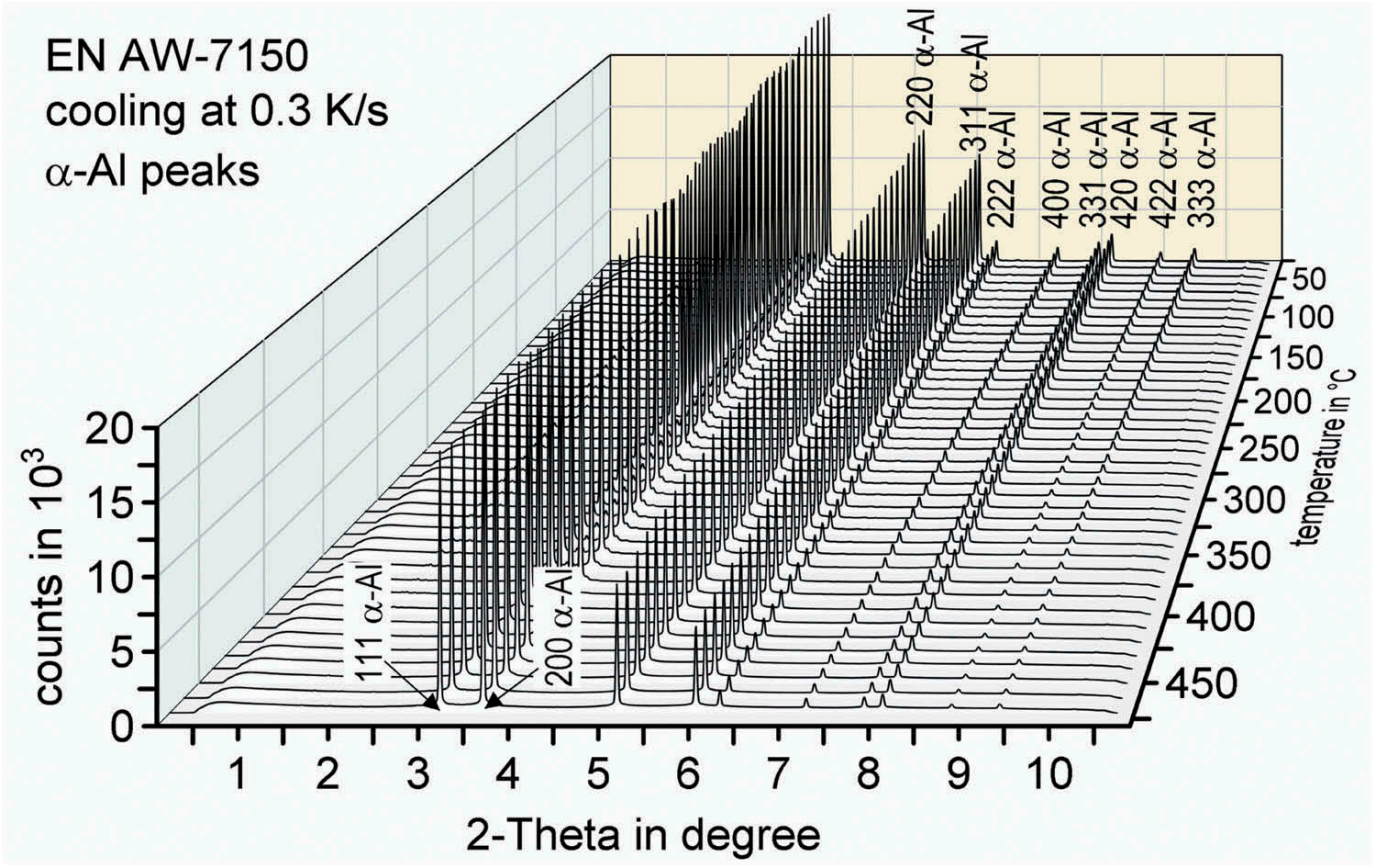
Dynamic evolution of diffraction pattern during the cooling of EN AW-7150 at 0.3 K/s to room temperature after solution annealing at 480°C

The evaluation of the diffractograms after integration was done using the analysis and graphing software Origin, developed by OriginLab Corporation. Figure 4 shows an overview of those diffractograms in the range of the investigated 2-Theta angles while cooling EN AW-7150 at 0.3 K/s after solution annealing at 480°C for selected temperature steps. It mainly shows the high intensity aluminium solid solution diffraction peaks.

**Figure 5.**
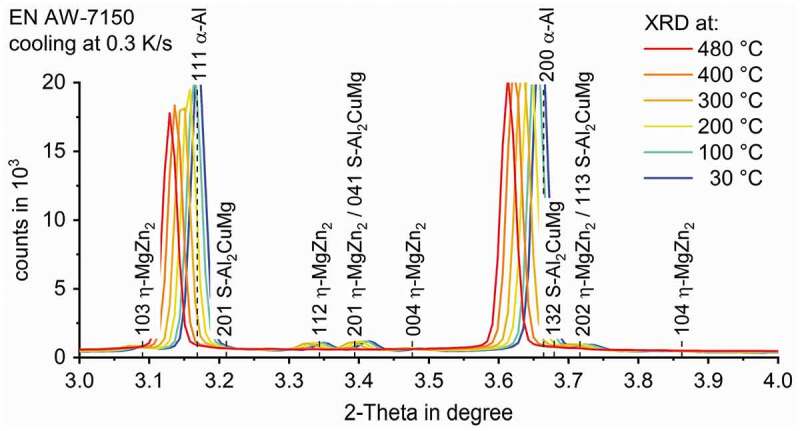
Detailed view of the diffraction pattern during the cooling of EN AW-7150 at 0.3 K/s at different temperatures showing the shift of high-intensity peaks

Figure 5 shows a detailed view of selected diffraction pattern during the cooling of EN AW-7150 at 0.3 K/s at certain temperatures. Each pattern is coloured from high-temperatures in red to low-temperatures in blue. The range of 2-Theta angles is limited around the strongest peaks for the relevant phases, η-MgZn_2_ and S-Al_2_CuMg. As can be seen in Figure 5, a shift of specific peaks to higher 2-Theta angles occurs with decreasing temperature. Shifts of diffraction peak are generally attributed to changes in the corresponding mean lattice distance [32]. Here it can be expected that the dominating effect leading to changes in the corresponding mean lattice distances is the temperature-dependent lattice shrinkage (related to thermal ‘expansion’ during cooling). For example, the 100% peak of the α-Al shifts from 3.12° at 480°C to 3.17° at 30°C. As a result of the temperature-dependent shift in combination with the proximity of distinct diffraction peaks (of different phases), certain peaks (including the 100% peaks for η-MgZn_2_ and S-Al_2_CuMg) are not usable for further evaluation due to superposition with another diffraction peak. Thus, for further evaluation, it is important to find the characteristic 2-Theta angles of the η-MgZn_2_ and S-Al_2_CuMg structures with high-intensity and sufficient distance to other specific peaks to avoid peak superposition at any temperature.

**Figure 6.**
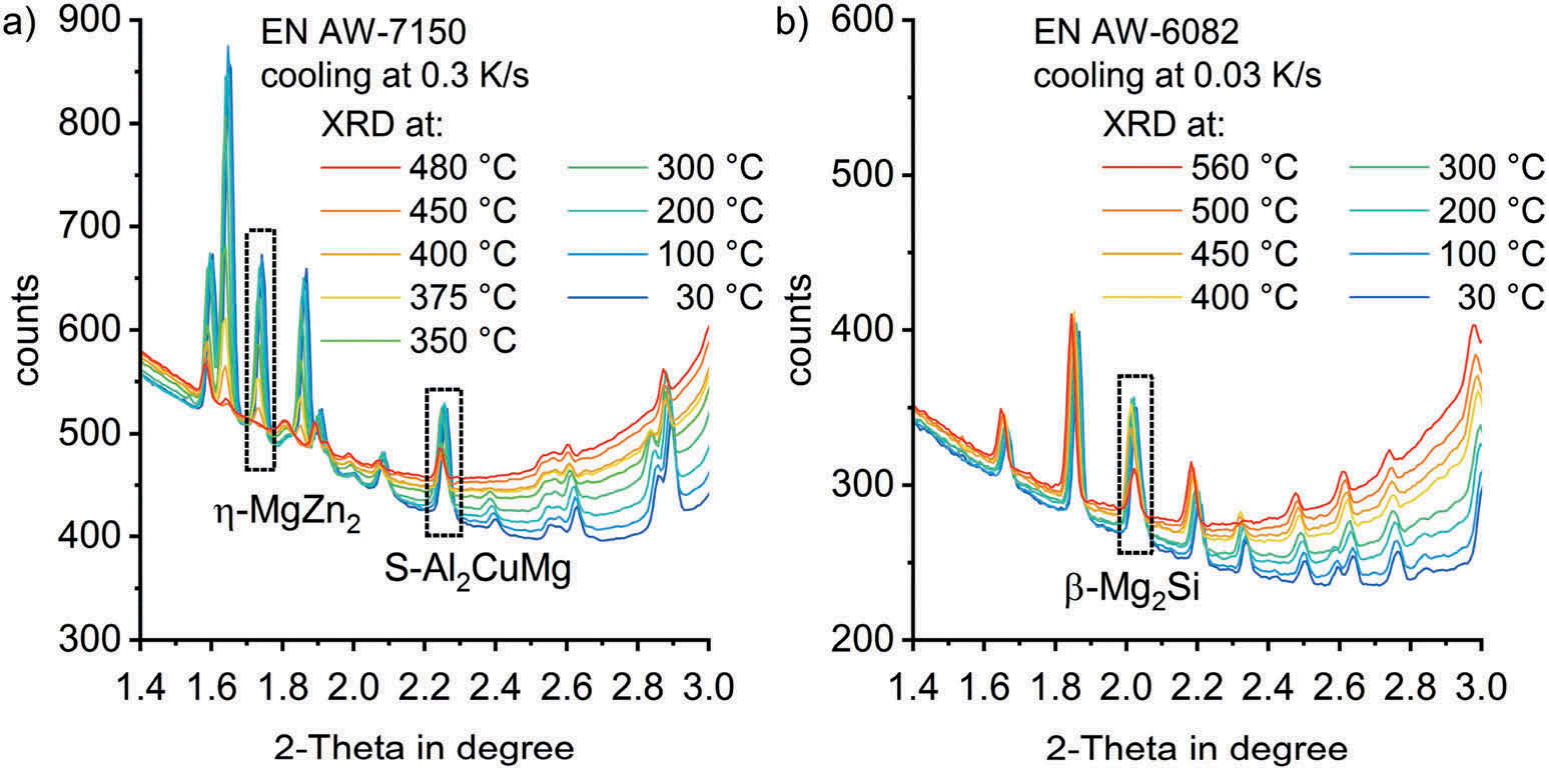
Detailed view of specific diffraction peaks for η-MgZn_2_ (1.73°), S-Al_2_CuMg (2.27°) (EN AW-7150 in a) and β-Mg_2_Si (2.02°) (EN AW-6082 in b) during cooling of a) EN AW-7150 at 0.3 K/s and b) EN°AW-6082 at 0.03 K/s. The background noise, which depends on 2-Theta angle and temperature can be seen

As shown in Figure 6 at lower 2-Theta angles, well separated characteristic peaks were found to be appropriate for evaluation. For EN AW-7150, the peaks labelled for the η-MgZn_2_ structure at 1.73° (002) and S-Al_2_CuMg at 2.27° (111) represent peaks with 33% and 60% intensity, respectively, see Figure 6(a). For the β-Mg_2_Si phase in EN AW-6082 the peak at 2.02° represents the diffraction peak with 41% with respect to the peak of highest intensity. The diffractograms at selected temperatures in Figure 6 give an idea of the dynamic evolution of the quench induced precipitation of the phase structures of η-MgZn_2_ and S-Al_2_CuMg in EN AW-7150 (Figure 6(a)) and β-Mg_2_Si in EN AW-6082 (Figure 6(b)). According to the diffraction pattern in Figure 6(a)) at 480°C, a complete dissolution of the η-MgZn_2_ phase was achieved during solution annealing since no peak is present at 1.73°. With decreasing temperatures, the solubility of alloying elements in the surrounding matrix decreases. Therefore, quench induced precipitation occurs between 450–400°C. At 400°C, a first small peak is visible at the 2-Theta angle corresponding to the η-MgZn_2_ structure. Further cooling leads to more pronounced precipitation while the peak reaches a maximum at 200°C, indicating that the precipitation has finished.

In contrast to η-MgZn_2_, S-Al_2_CuMg was not completely dissolved during solution annealing. In the diffraction pattern at 480°C at the beginning of the cooling process, there is a clear peak at 2.27°corresponding to S-Al_2_CuMg particles. This observation is in agreement with other research with respect to remaining particles after the solution annealing of EN AW-7150 [38]. With decreasing temperature, precipitation of this phase occurs between 375–350°C. A maximum is reached at about 200°C, which indicates the end of the precipitation. Similar can be stated on β-Mg_2_Si in EN AW-6082 (Figure 6(b)), although the solution treatment here was meant to dissolve all β-Mg_2_Si particles [10].

### 3.3 Background subtraction and signal normalisation allowing quantitative comparison of peaks with different diffraction intensities

Figure 6 shows a significant background noise depending on 2-Theta angle and temperature. It becomes clear that there is a need for separately normalising each peak at each temperature step to compare the diffraction patterns at different time/temperature steps and enable the comparison of different phases.

**Figure 7.**
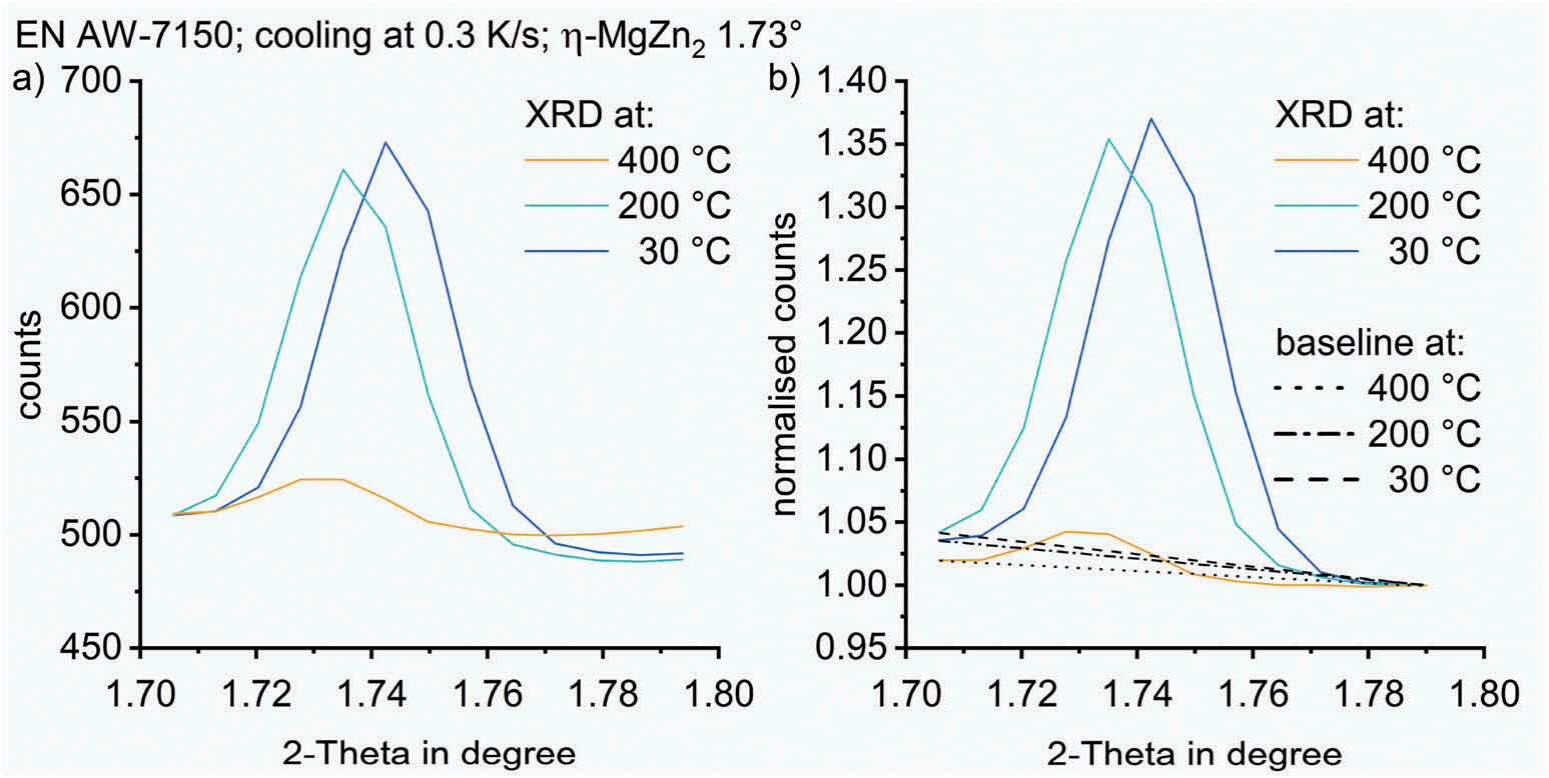
(a) detected diffraction counts for the 002 distance of the η-MgZn_2_ structure formation in EN AW-7150 during cooling at 0.3 K/s for selected temperatures; (b) normalised signal step specific baselines used for peak area integration

To normalise the η-MgZn_2_ structure peak at 1.73°, the data is limited to 2-Theta angles ranging from 1.70–1.78°, covering the whole peak width and taking the temperature-shift into account, see Figure 7. To evaluate the time/temperature-dependent precipitation of the S-Al_2_CuMg phase structure, data separation was applied to the data ranging from 2.21–2.30°.

**Figure 8.**
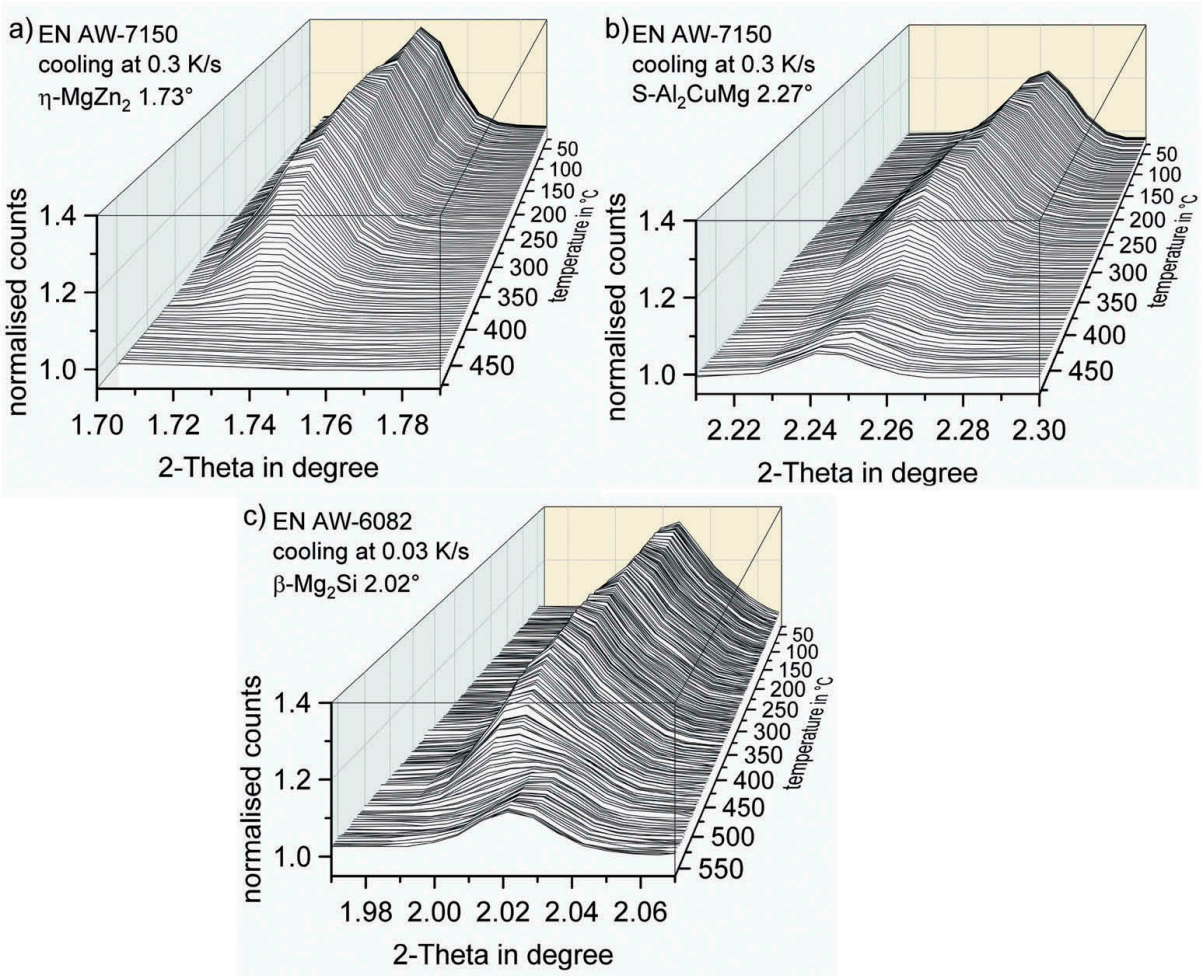
Dynamic evolution of normalised WAXS peaks for EN AW-7150 during cooling at 0.3 K/s after solution treatment for (a) the η-MgZn_2_ structure (002, 1.73°), (b) the S-Al_2_CuMg phase structure (111, 2.27°) and (c) the β-Mg_2_Si structure (111, 2.02°) during cooling of EN AW-6082 at 0.03 K/s

The normalisation of specific peaks needs to be done using the counts of a 2-Theta value without any peak, ideally next to the specific peak for each temperature. The counts at 1.79° were used to normalise the data range for η-MgZn_2_. This procedure is illustrated in Figure 7. The counts at 2.21° were used for normalisation the S-Al_2_CuMg phase (111) peak (at room temperature nominally at 2.27°). The temperature-dependent evolution of the selected WAXS peaks are plotted in Figure 8(a–c). Figure 8 clearly shows that all peak areas increase during cooling, i.e. precipitation takes place.

The normalised peaks areas have been integrated for quantitative comparison. As an integration boundary, straight lines were defined between the endpoints of the selected normalised diffraction peaks at each temperature step (see Figure 7(b)).

**Figure 9.**
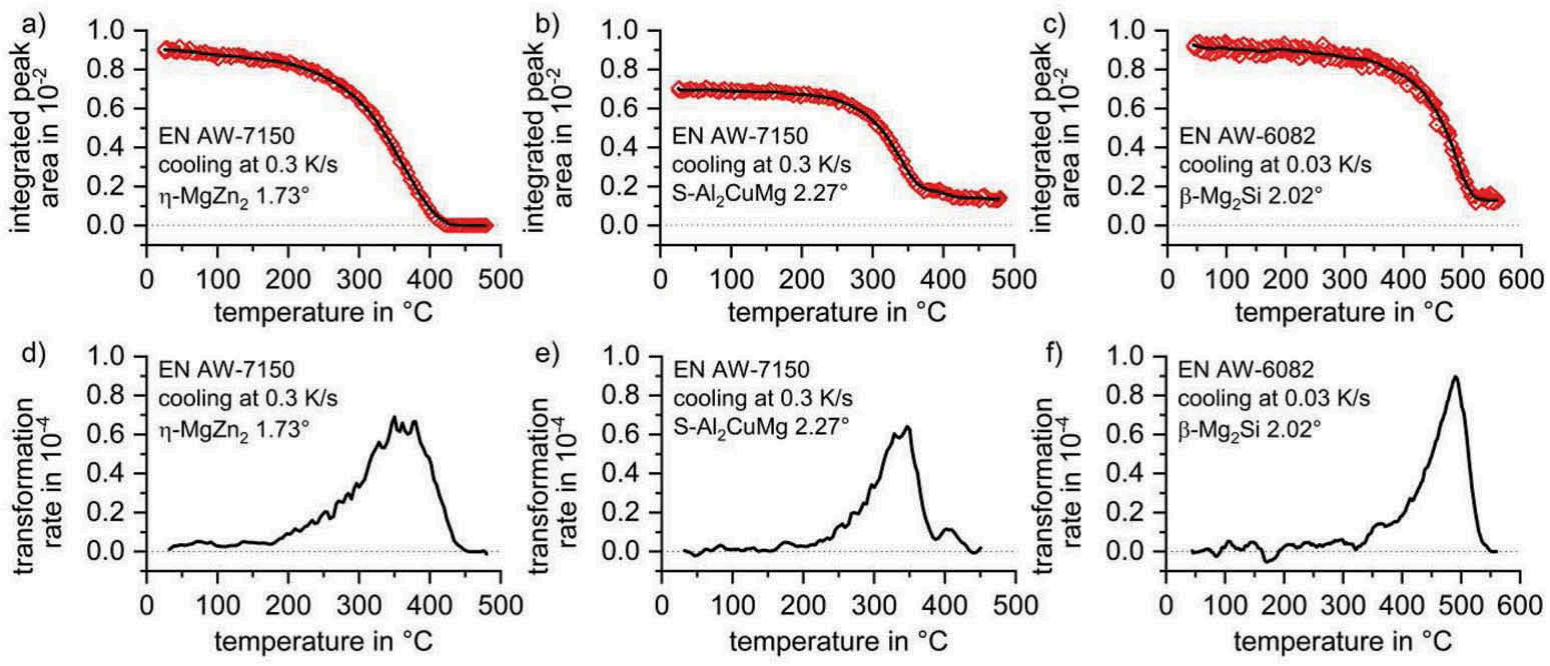
(a), (b), (c) integrated peak areas of η-MgZn_2_, S-Al_2_CuMg and β-Mg_2_Si; (d), (e), (f) differentiation of the smoothed integral curve to the process variable ‘transformation rate’

As shown in Figure 9(a–c), the data points of the integrated peak areas at the discrete temperature steps (red symbols) were transformed to one continuous curve using the moving average smoothing approach. Because the normalisation the continuous curve of the integrated WAXS-peak areas as a function of temperature is proportional to the precipitated phase fraction, they are, thus, comparable to the state variable ‘precipitation enthalpy’ measured by DSC. Therefore, a derivation of that curve allows transforming the state variable into a process variable ‘transformation rate’, Figure 9(d–f), which is comparable to the excess specific heat capacity in DSC curves.

In EN AW-7150 the η-Mg(Zn,Cu,Al)_2_ phase was completely dissolved during solution annealing. From Figure 9(a,d), it is apparent that the precipitation of a phase fraction with the η-MgZn_2_ structure starts at 450°C while the maximum of transformation rate is reached at 375°C. The precipitation of η-Mg(Zn,Cu,Al)_2_ during cooling at 0.3 K/s was finished at about 180°C. Not all S-Al_2_CuMg phase particles were dissolved during solution annealing as its (111) plane caused a diffraction signal even at solution treatment temperature, see Figure 8(b). The further precipitation of S-Al_2_CuMg started at 440°C while the maximum transformation rate was reached at 350°C, see Figure 9(b,e). The precipitation reaction of the S-Al_2_CuMg phase in EN AW-7150 finished at around 200°C. From Figure 6(b) and Figure 8(c) it appears that by the solution treatment of this batch of EN AW-6082 the phase β-Mg_2_Si was not fully dissolved. But still precipitation during cooling first starts after a slight undercooling at about 550°C, see Figure 9(c,f). The β-Mg_2_Si transformation rate according to WAXS has its maximum at about 490°C, while the precipitation of β-Mg_2_Si seems to be finished at about 300°C.

## 4 Correlation of the continuous cooling WAXS signals to cooling DSC

### 4.1 Alloy EN AW-7150

**Figure 10.**
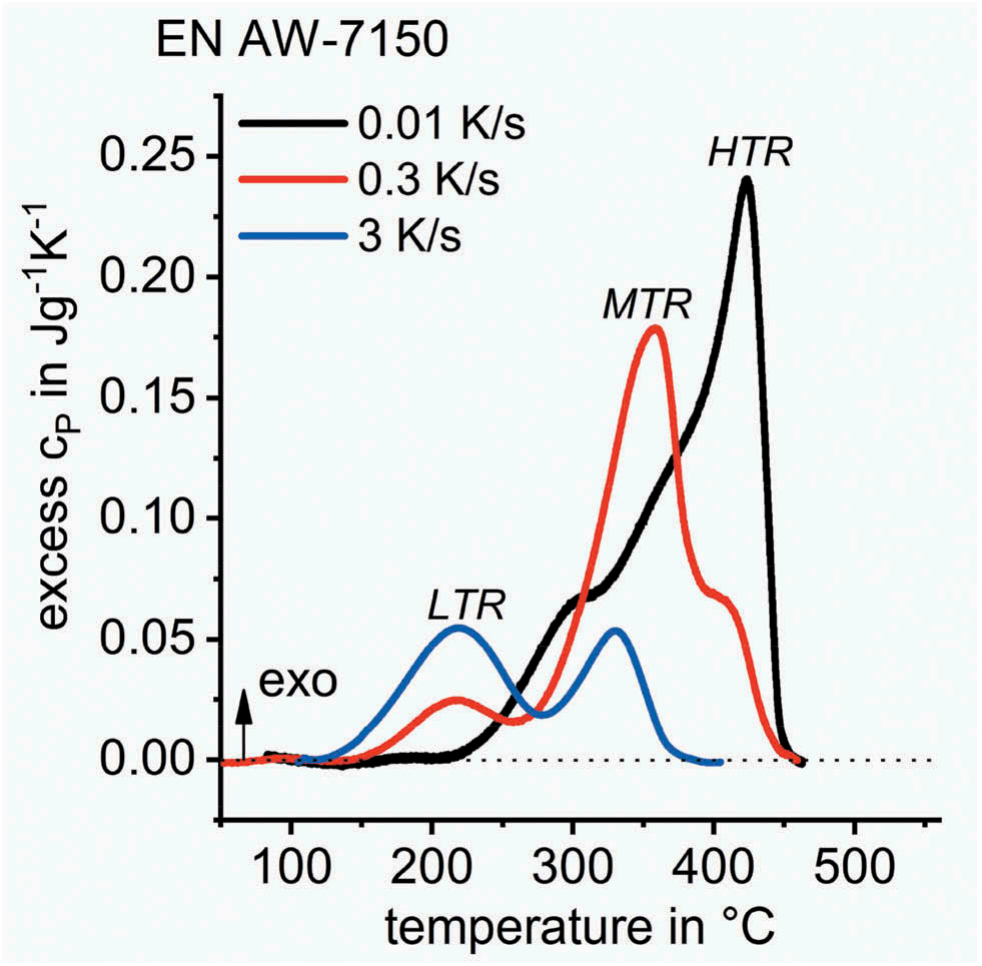
DSC cooling curves of EN AW-7150 after solution treatment indicating a minimum of three precipitation reactions; DSC data adapted from [8]

Figure 10 shows DSC cooling curves as a function of temperature for Al-Zn-Mg-Cu alloy EN AW-7150 indicating multiple quench induced precipitation reactions. Deviations of the measured signal exceeding the zero level indicate exothermic precipitation reactions. Figure 10 demonstrates the dynamic/kinetic evolution of quench induced precipitation for this alloy in a broad range of cooling rates.

For EN AW-7150 cooling, DSC shows at least three main reactions – high-temperature reactions (HTR), medium-temperature reactions (MTR) and low-temperature reactions (LTR). The overlapping of HTR and MTR is severe and separating these two peaks seems impossible based on DSC. Previous studies have claimed that those two reactions refer to the precipitation of the S-Al_2_CuMg and η-Mg(Zn,Cu,Al)_2_ phases [8,16], where precipitation of S-Al_2_CuMg was assigned to the HTR and precipitation of η-Mg(Zn,Cu,Al)_2_ was associated to the MTR. The overlapping of any of the above discussed reactions kinetically changes with changing cooling rate. Though the overlapping has never been specifically quantified, neither in terms of precipitation start/end temperatures nor in terms of the related precipitation enthalpies (peak areas) within the overlapping temperature region. Thus, for evaluating continuous cooling precipitation diagrams, so far the relevant precipitation temperature regions have been estimated only using the low- or saddle-point between two peaks or a peak and its shoulder, as specified in [8,17].

**Figure 11.**
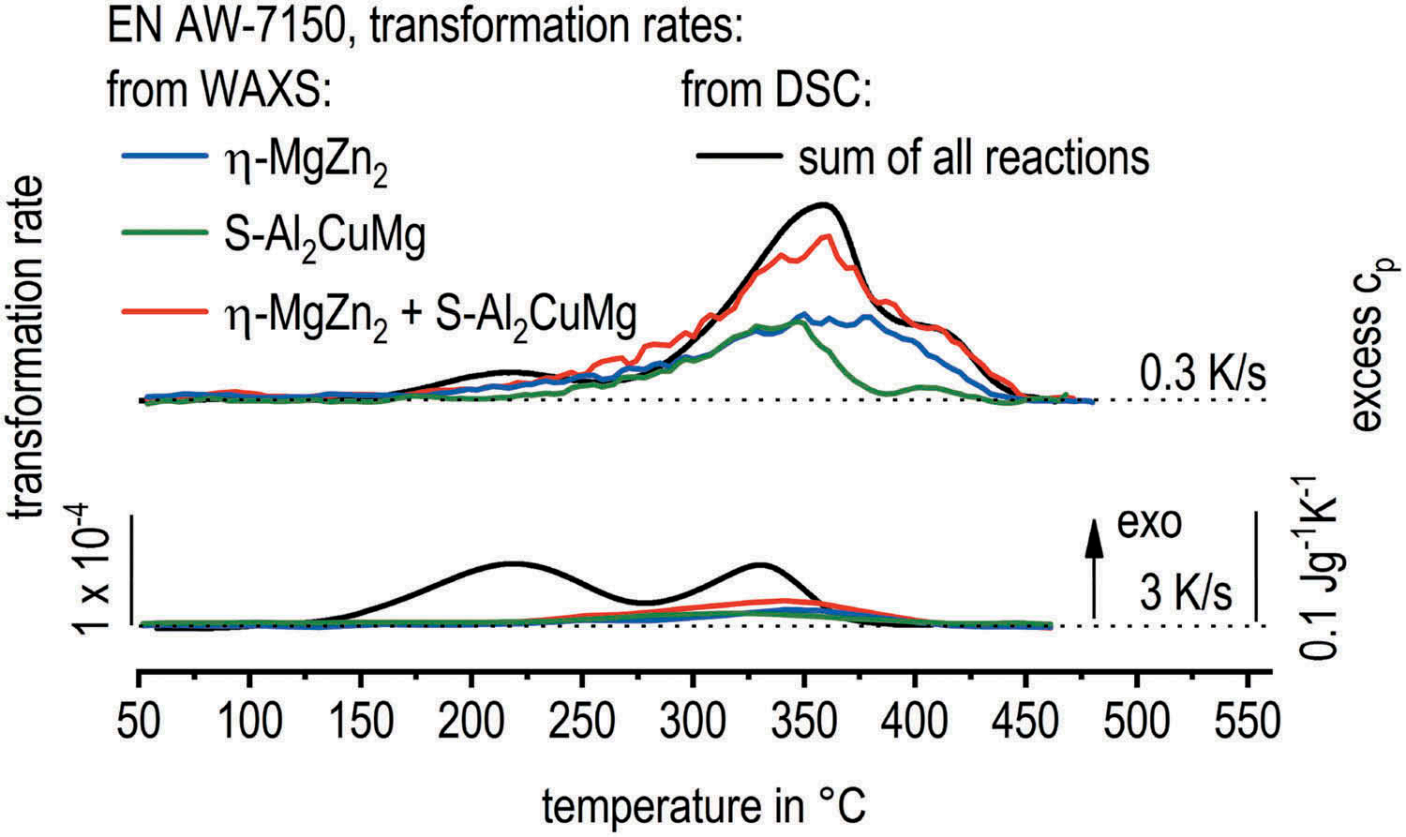
Comparison of obtained WAXS results to DSC curves [8] during cooling of EN AW-7150 after solution treatment

Figure 11 presents the major result of this work for EN AW-7150 comparing the transformation rates obtained for certain quench induced phases from continuous cooling in situ WAXS with DSC cooling curves. The curves of different cooling rates are shifted above each other to improve readability, starting with the lowest cooling rate on top. The WAXS signal is plotted in arbitrary units and the scaling was adjusted using the 0.3 K/s cooling rate so that the WAXS signal height is equivalent to the DSC signal height at certain temperatures. For both alloys, one scaling factor was defined and kept constant for all cooling rates. For each cooling rate, a corresponding zero-level is plotted as a dotted line. Deviations of the measured signals exceeding this zero level indicate precipitation reactions for both methods used.

Figure 11 correlates the distinct transformation rates for η-MgZn_2_ (blue) and S-Al_2_CuMg (green) from WAXS with DSC cooling curves of EN AW-7150 (black), which equals the sum of transformation rates for all contributing phases. The WAXS results confirm that both phase structures, η-MgZn_2_ and S-Al_2_CuMg, contribute to the process of quench induced precipitation, as reported in the literature [6,16]. For improved comparison to the DSC signal, the sum of the WAXS transformation rates for the η-MgZn_2_ and S-Al_2_CuMg structures was plotted (red). This sum shows a good fit to the DSC signal, particularly for 0.3 K/s and temperatures relevant for the precipitation of η-MgZn_2_ and S-Al_2_CuMg. This indicates, that indeed these two phases contribute to the HTR and MTR seen in DSC. Though, unlike assumed in the literature, the WAXS results indicate that the precipitation temperature range for η-Mg(Zn,Cu,Al)_2_ is very broad, covering the temperature ranges of both HTR and MTR from DSC. Precipitation of the η-Mg(Zn,Cu,Al)_2_ phase seems to start with the total onset of precipitation upon cooling. Moreover, for 0.3 K/s, WAXS indicates that the MTR-peak in DSC is related to the precipitation of S-Al_2_CuMg, rather than only to η-Mg(Zn,Cu,Al)_2_ as assumed previously. Thereby WAXS shows that the superposition of these two precipitation reactions is more severe than expected. This seems reasonable since both phases compete on the same alloying elements and thus local concentration and nucleation sites might determine the type of crystal structure to which a certain particle tends to precipitate. At 3 K/s, the LTR around 200°C could not be detected by WAXS. This is due to the small size of the LTR precipitates [9].

### 4.2 Alloy EN AW-6082

**Figure 12.**
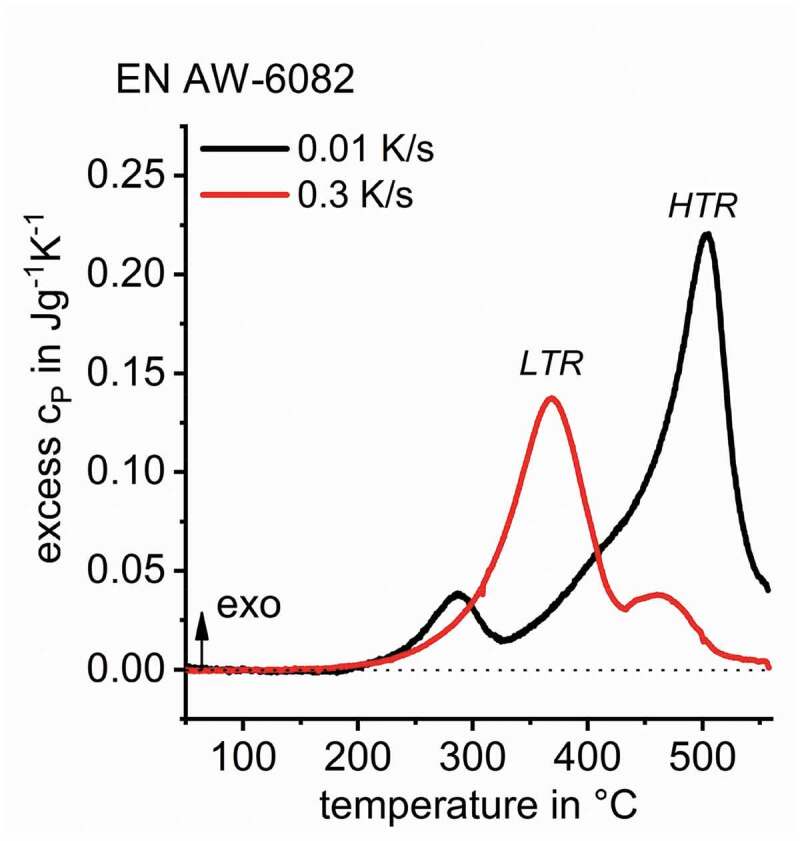
DSC cooling curves for EN AW-6082 after solution treatment, indicating a minimum of two precipitation reactions; DSC data adapted from [10]

For EN AW-6082, in Figure 12, DSC shows at least two partially overlapping precipitation reactions – HTR and LTR. Previous studies found the HTR to be predominantly related to the precipitation of β-Mg_2_Si with particle dimensions of a few tens of µm down to some hundreds of nm [11,39]. The LTR is predominantly related to the precipitation of metastable B’-Mg_5_Si_4_Al_2_ with particle dimensions of a few hundreds of nm down to a few tens of nm [11,17].

**Figure 13.**
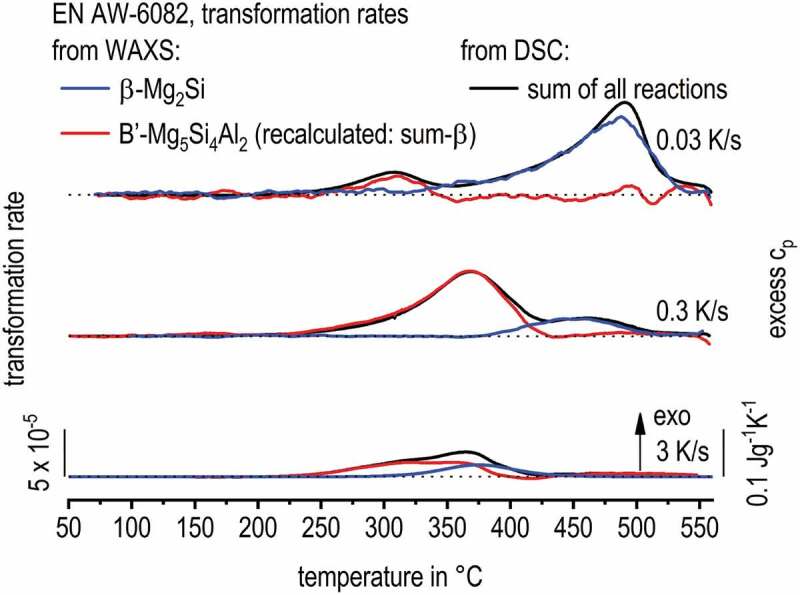
Comparison of WAXS results to DSC curves [10] during the cooling of EN AW-6082 after solution treatment

For WAXS evaluation of EN AW-6082, the same method as for EN AW-7150 was used. To investigate the kinetics of fcc β-Mg_2_Si, the peak at a 2-Theta angle of 2.02°, corresponding to the (111) plane, was used. The DSC and WAXS data of EN AW-6082 is correlated in Figure 13, presenting the major result of this work for this alloy. The WAXS signal of β-Mg_2_Si shows a very good fit to the high-temperature peak detected by DSC, which particularly holds for 0.03 and 0.3 K/s. As with DSC, the WAXS-peak areas also decrease with increasing cooling rate. This indicates a reduction of the precipitate volume fraction with increasing cooling rate, which is reasonable due to the kinetic suppression of quench induced precipitation. For 3 K/s, in DSC, the peak superposition increased substantially and just one broad precipitation peak is seen. Former work (SEM, EDX, EBSD & XRD) showed that the precipitation of β-Mg_2_Si refers to the HTR [39]. This finding is confirmed by the in situ WAXS data. From previous work, it is moreover assumed that the LTR is related to B’-Mg_5_Si_4_Al_2_ [17]. Unfortunately, for the B’-Mg_5_Si_4_Al_2_ phase in the database, no diffraction pattern is known and diffraction peaks could not be assigned. To enable further deconvolution of reaction superposition, we assume, that in EN AW-6082 precipitation during continuous cooling is limited to the two phases β-Mg_2_Si and B’-Mg_5_Si_4_Al_2_. If so, the excess specific heat capacity (excess c_p_) signal from DSC is the sum of transformation rates for these two phases. By the evaluated and manually scaled transformation rate obtained by WAXS for the β-Mg_2_Si phase we now know one term of the sum. Thus we take the sum (excess c_p_ from the DSC) and subtracted the β-Mg_2_Si transformation rate to recalculate the virtual transformation rate for the B’-Mg_5_Si_4_Al_2_ phase. For quantitative evaluation of the WAXS intensities towards phase volume fractions, the R-value (relation of diffraction intensity to phase fraction) would be required [40]. The R-value is unknown for β-Mg_2_Si.

**Figure 14.**
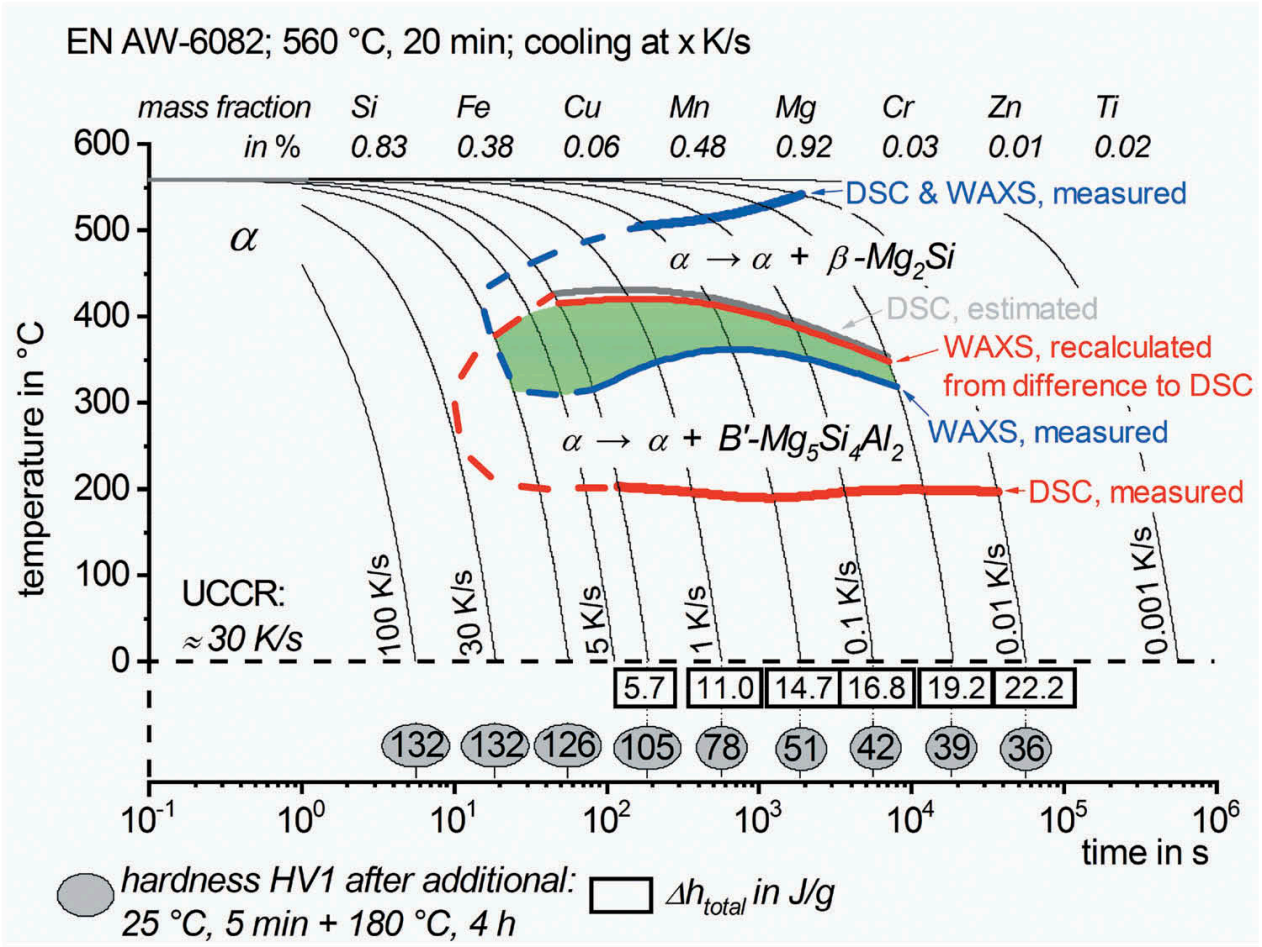
Improved continuous cooling precipitation diagram of EN AW-6082 after 560°C for 20 min, indicating the temperature ranges of overlapping precipitation reactions

As another major result Figure 14 shows a substantially improved version of the continuous cooling precipitation diagram for a given composition of EN AW-6082. The obtained WAXS transformation rates for the precipitation of β-Mg_2_Si and the recalculated signal for B’-Mg_5_Si_4_Al_2_ were used to determine the characteristic precipitation start- and end-temperatures in regions of overlapping reactions, which is highlighted in green. The upper critical cooling rate was estimated from the saturation hardness after ageing and was about 30 K/s [10]. The precipitation regions thus have been extrapolated based on the hardness results.

## 5 Conclusions

WAXS allows the identification of crystal structures. Combining short WAXS measurement times (ensuring sufficient counts by using synchrotron X-ray sources) with a heat treatment, the in situ detection of solid-solid phase transformations (e.g. precipitation) becomes possible.

The objective of this work is to improve the analysis of precipitation reactions during continuous cooling and particularly the separation of single reactions. Often DSC is used for the kinetic analysis of precipitation. However, DSC has the disadvantage to measure the heat effects of any current phase transformation as a sum. In this work precipitation during continuous cooling was analysed via in situ cooling WAXS, which gives information on the presence of single phases and thus allows the intended separation of single reactions. Cooling experiments were performed at 0.03 to 3 K/s with single WAXS diffraction patterns in discrete temperature steps of 1 to 15 K.

To enable the direct comparison of the WAXS and DSC signals and quantitative WAXS data evaluation, a sophisticated data treatment was required, including the following:

▪ normalisation of the WAXS signal for quantitative diffraction peak comparison
▪ integration of the normalised WAXS diffraction peaks areas with respect to 2 Theta reveals a state variable that is proportional to the precipitation enthalpy or precipitation volume fraction
▪ differentiation of the normalised WAXS diffraction peaks areas with respect to temperature transforms the state variable to a temperature-dependent process variable, which is directly comparable to the DSC signal (excess specific heat capacity) since both signals indicate the transformation rate at a certain temperature.

In this work, in situ cooling WAXS was applied successfully to the detection of quench induced precipitation of the β-Mg_2_Si phase in an Al-Mg-Si alloy EN AW-6082 and for the detection of the η-MgZn_2_ and S-Al_2_CuMg phases in an Al-Zn-Mg-Cu alloy EN AW-7150. The obtained WAXS data substantially improved the knowledge base about the superposition of different phase precipitations. The following can be concluded for the single alloys and phases:

▪ For EN AW-6082, the previously existing interpretation of the DSC curve was correct and WAXS confirmed, that the precipitation of β-Mg_2_Si dominates the high temperature reaction peak seen in DSC. Beyond that the temperature interval, in which the precipitation of β-Mg_2_Si and B’-Mg_5_Si_4_Al_2_ overlap was determined and, thus, enabled updating the continuous cooling precipitation diagram of EN AW-6082.
▪ For EN AW-7150 the previously existing assignment of precipitation reactions to the DSC curves assumed consecutive reactions with a limited overlap. WAXS now showed that the overlap is severe and precipitation of η-MgZn_2_ happens in a very broad temperature range contributing both high and medium temperature reactions seen in DSC. Other than expected so far, WAXS revealed that the medium temperature reaction is mainly due to precipitation of the S-Al_2_CuMg phase.
